# Oncostatin M is dispensable for the regulation of hematopoietic stem/progenitor cell traffic by neutrophils

**DOI:** 10.1016/j.isci.2025.112646

**Published:** 2025-05-12

**Authors:** Anna Rodella, Carlotta Boscaro, Francesco Ivan Amendolagine, Ludovica Migliozzi, Barbara Molon, Antonella Viola, Mattia Albiero, Gian Paolo Fadini

**Affiliations:** 1 Veneto Institute of Molecular Medicine, 35100 Padova, Padua, Italy; 2 Department of Medicine, University of Padova, 35100 Padova, Padua, Italy; 3 Department of Biomedical Sciences, University of Padova, 35128 Padua, Italy; 4 Department of Surgery, Oncology and Gastroenterology, University of Padova, 35100 Padua, Italy

**Keywords:** Immunology, Components of the immune system

## Abstract

Hematopoietic stem/progenitor cell (HSPC) trafficking in and out of the bone marrow (BM) is essential for immune surveillance and hematopoietic balance. We previously identified Oncostatin M (OSM), primarily from myeloid cells, as a key regulator of HSPC traffic. Here, we show that neutrophils highly express and secrete OSM, especially when senesced. However, OSM is not required for neutrophil-mediated modulation of steady-state or circadian HSPC levels. Aged neutrophils returning to the BM reduce HSPC levels in peripheral blood (PB) independently of OSM, suggesting additional mechanisms beyond CXCL12/CXCR4 axis. While neutrophil transfer modulated HSPC kinetics in wild-type mice, OSM-secreting neutrophils failed to normalize elevated PB-HSPC levels in *Osm*^−/−^ mice, though recombinant OSM successfully did. Macrophage depletion-induced HSPC egress was OSM-dependent, but neutrophil depletion elevated PB-HSPCs regardless of OSM. These findings reveal that neutrophils regulate HSPC migration via largely OSM-independent pathways, emphasizing the importance of cell-specific and context-dependent cues within the BM niche.

## Highlights

• OSM is highly expressed and released by activated neutrophils, especially if senescent
• Loss of OSM persistently elevated blood HSPCs without affecting neutrophil kinetics
• OSM secretion from neutrophils does not regulate HSPCs kinetics in/out their niche
• Neutrophil depletion mobilizes HSPCs independently from OSM and CXCL12/CXCR4

## Graphical abstract

**Figure:**
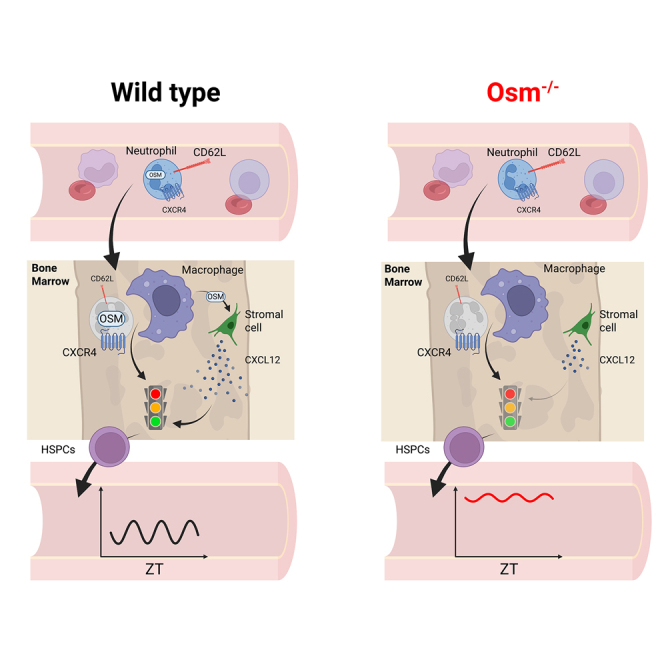

## Introduction

The traffic of hematopoietic stem/progenitor cells (HSPCs) in and out the bone marrow (BM) is a finely tuned process that tightly regulates the levels of HSPCs in peripheral blood.^1^ In turn, peripheral HSPCs contribute to immune surveillance and hematopoietic homeostasis.^2^ Notably, a disruption in the levels of circulating HSPCs can be observed with aging^3^ and in the setting of hematologic disorders (e.g., myelofibrosis) or non-hematologic conditions (e.g., diabetes mellitus).^4^ In addition, low HSPCs are associated with poor outcomes and excess mortality, especially for cardiovascular causes.^5^ Thus, unveiling the mechanisms that govern HSPC trafficking may enable strategies to improve immune and hematopoietic efficiency in these conditions. In particular, the circadian fluctuation of HSPC levels in the bloodstream is thought to ensure the synchronization with immune cell function, patrolling of peripheral lymphoid organs, and hematopoietic activity.^6^

We and others have unveiled that Oncostatin M (OSM) is a BM niche factor produced by myeloid cells and involved in the regulation of BM-HPSC retention versus release.^7^^,^^8^ OSM secreted by CD169^+^ M1-like macrophages in the BM retains HSPCs within the niche by inducing CXCL12 in stromal cells.^9^ In addition, OSM may intrinsically attenuate HSPC chemotactic response to CXCL12 and increase their adhesion to the BM stroma by inducing E-selecting on OSMR^+^ BM endothelial cells.^9^ In unstimulated conditions, *Osm*^−/−^ mice display markedly elevated HSPCs in peripheral blood, suggesting that OSM is required for HSPCs returning to the BM. Consistently, the homing and engraftment of transplanted HSPCs improves with injection of recombinant OSM in recipient mice.^10^ At the same time, homing of HSPCs to the BM is regulated by the rhythmic clearance of aged neutrophils.^11^ Maintaining a pool of neutrophils, which are abundant and short-lived, requires a coordinated recycling in the BM. Removal of neutrophils from the blood is instrumental to sensing hematopoietic needs and is therefore linked to HSPC dynamics. Cyclic elimination of aged CD62L^low^CXCR4^high^ neutrophils in the BM elicits signals that regulate the HSPC niche and determines blood HSPC levels. Such signals have been incompletely understood.

Starting from the observation that neutrophils have high *Osm* expression and release OSM, especially when aged, we examined whether OSM produced by senescent neutrophils that return to the BM affects the niche as a molecular brake to HSPC release, thus reducing HSPCs in peripheral blood.

## Results

### Oncostatin M is expressed and released by neutrophils

**Figure 1:**
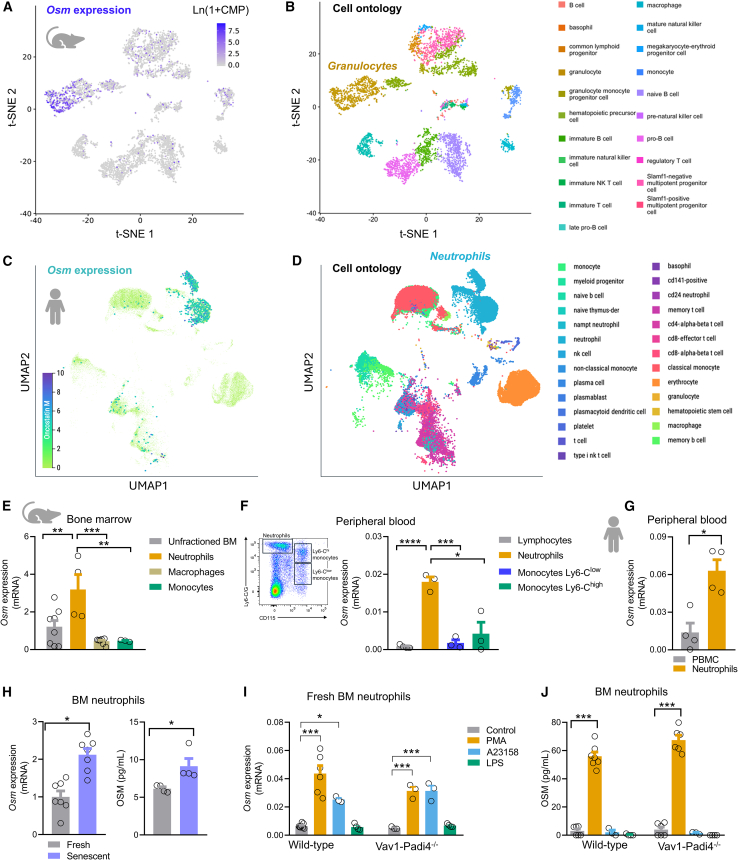
Expression and release of OSM by neutrophils (A) Single cell gene expression analysis of *Osm* in mouse hematopoietic cell populations. (B) Corresponding spatial gene ontology showing overlap of neutrophils with *Osm* expression. (C) Single cell gene expression analysis of *Osm* in human hematopoietic cell populations. (D) Corresponding spatial gene ontology showing overlap of neutrophils with *Osm* expression. (E and F) *Osm* gene expression in cell populations of the murine bone marrow (E) and peripheral blood (F). (G) *Osm* gene expression in neutrophils versus unfractionated peripheral blood mononuclear cells (PBMCs) from healthy human blood donors. (H) Expression of *Osm* (left) and secretion of OSM in the medium (right) in neutrophils freshly isolated from the bone marrow (BM) in unstimulated condition or stimulated with PMA. (I) Secretion of OSM in the medium by fresh or senescent BM-derived neutrophils. (J) Secretion of OSM in the medium by freshly isolated BM-derived neutrophils of wild-type or NETosis-deficient Padi4^−/−^ mice in unstimulated condition, stimulated with PMA or with NETosis-inducing agents A23158 ionophore and LPS. Mean ± standard error is displayed in the plots. ∗*p* < 0.05; ∗∗*p* < 0.01; ∗∗∗*p* < 0.001; ∗∗∗∗*p* < 0.0001 by Student’s *t* test (G, H) or by one-way ANOVA (E, F, I and J). n = 3–8 biological replicates for each strain.

We first examined publicly available single-cell gene expression profiles^12^^,^^13^ to track *Osm* in murine BM cell types and in human blood cell types and found that *Osm* segregates with mouse granulocytes (Figures 1A and 1B) and human neutrophils (Figures 1C and 1D). Given that macrophages are typically under-represented in *tabula muris* and, in general, in scRNA-seq analyses,^14^^,^^15^ we wanted to confirm this finding in C57Bl/6 mice: in the BM, *Osm* gene expression was >2-fold higher in neutrophils than in monocytes or macrophages (Figure 1E) and, in the PB, *Osm* gene expression was >3-fold higher in CD115^-^ Ly6-G^high^ neutrophils than in lymphocytes, and in CD115^+^Ly6-C^high^ or CD115^+^Ly6-C^low^ monocytes (Figure 1F). Using human blood samples, we confirmed that *OSM* gene expression was >3-fold higher in fresh purified neutrophils than in PBMCs (Figure 1G).

Mouse neutrophils freshly isolated from the BM and senesced *in vitro* for 16 h stained positive for the senescence marker beta-galactosidase (Figure S1) and had significant *Osm* upregulation and OSM secretion in the medium (Figure 1H). When stimulated with PMA or A23128 ionophore, canonical inducers of NETosis, freshly isolated murine BM neutrophils displayed an increase in *Osm* transcription (Figure 1I), but OSM release in the medium was detected only after PMA stimulation (Figure 1J). LPS stimulation exerted no effects on either *Osm* transcription or release of OSM. Of note, this pattern was preserved in neutrophils isolated from NETosis-deficient *Vav1-Padi4*^−/−^ mice (Figures 1I and 1J). Annexin V/Propidium iodide (PI) staining revealed that PMA induced a raise in putative necrotic cells (AnnV^−^PI^+^ cells) and of late-apoptotic cells (AnnV^+^PI^+^ cells; Figure S2) in both Padi4KO and Wt neutrophils. Conversely, Padi4KO neutrophils stimulated with A23158 ionophore were protected from early apoptosis (AnnV^+^PI^−^) compared to Wt neutrophils (Figure S2). While dissecting neutrophil cell death by apoptosis, necrosis, or NETosis can be challenging, it seems that PMA can activate *Osm* transcription in neutrophils, but whether OSM is actively secreted or passively released upon cell death remains unclear. Altogether, these data indicate that *Osm* is expressed by neutrophils, especially when senescent, more than by other hematopoietic cells in the PB and BM. Neutrophil activation results in the release of OSM, which is unrelated to NETosis.

### Loss of OSM leads to persistently elevated blood HSPCs without affecting neutrophil kinetics

**Figure 2:**
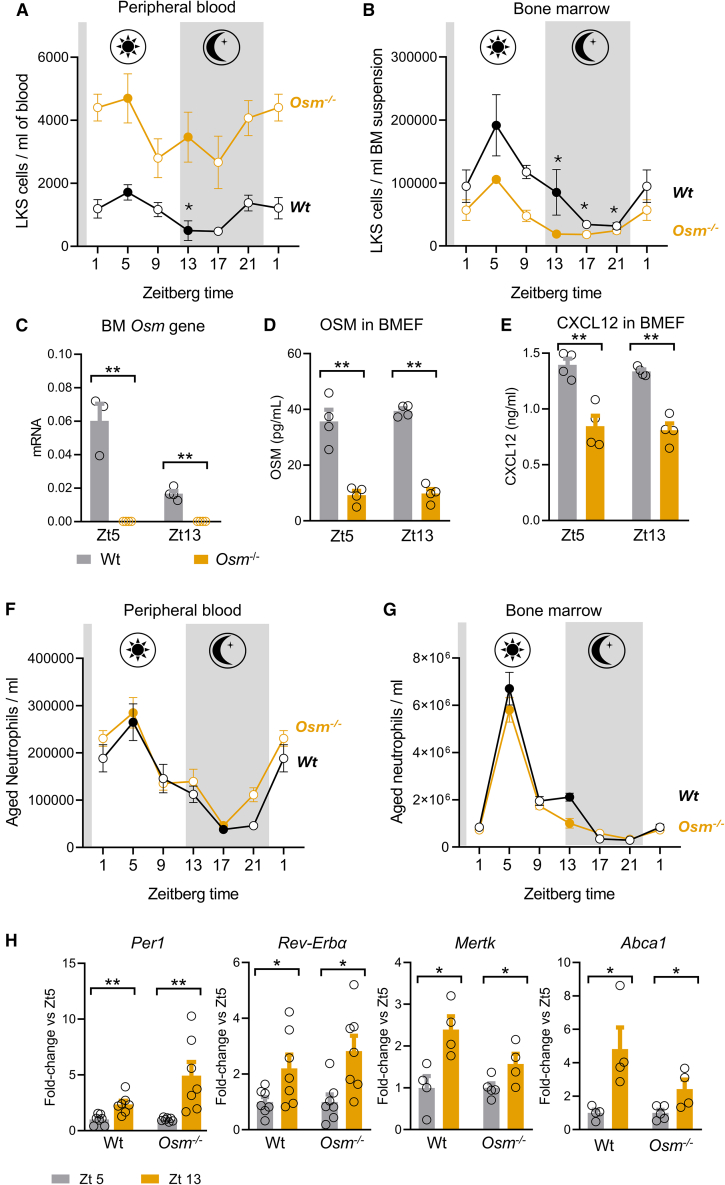
Loss of OSM perturbs HSPC levels but not neutrophil level and circadian rhythms (A and B) Circadian levels of HSPCs (Lin^-^cKit^+^Sca1^+^ cells, LKS) in peripheral blood (A) and bone marrow (B) of wild-type (Wt) and *Osm*^−/−^ mice at different time points. Asterisks report significance vs. ZT5 of each chart. (C and D) *Osm* gene expression (C) and OSM concentration in the bone marrow extracellular fluid (BMEF, D) in Wt and *Osm*^−/−^ mice. (E) CXCL12 concentrations in the BMEF in Wt and *Osm*^−/−^ mice. (F and G) Circadian levels of aged neutrophils in peripheral blood (F) and bone marrow (G) of Wt and *Osm*^−/−^ mice at different time points. (H) Expression of selected clock genes in neutrophils and of phagocytosis genes in the BM of Wt and *Osm*^−/−^ mice at Zt5 and Zt13, normalized to Ubc. Mean ± standard error is displayed in the plots. ∗*p* < 0.05; ∗∗*p* < 0.01; by Student’s *t* test (A, B, F, G, H), two-way ANOVA (C, D, E). n = 4–7 biological replicates for each strain.

In wild-type mice, levels of PB-HSPC, defined as LKS (Lin^-^cKit^+^Sca1^+^) cells, peaked at ZT5 (murine inactivity time) and reached a nadir at ZT13-17 (murine activity time, Figure 2A). A parallel circadian fluctuation of HSPC levels was observed in the BM (Figure 2B). In *Osm*^−/−^ mice, HSPC levels in peripheral blood remained significantly elevated throughout activity and inactivity time, never reaching down the levels seen in Wt mice (Figure 2A). Though the circadian difference (ZT5 vs*.* ZT13) was not statistically significant within the *Osm*^−/−^ group of mice, two-way ANOVA showed effects of time (*p* = 0.02) and of genotype (*p* < 0.0001) on HSPC levels, but not an interaction time-by-genotype (*p* = 0.71), suggesting a similar circadian trend in PB-HSPCs. *Osm*^−/−^ mice displayed a lower HSPC content in the BM, with a circadian trend similar to Wt mice (Figure 2B). In addition to absence of *Osm* gene transcript (Figure 2C) and very low concentrations of OSM protein in the BM extracellular fluid (BMEF, Figure 2D), *Osm*^−/−^ mice also displayed reduced concentrations of CXCL12 in the BMEF (Figure 2E), consistent with the notion that OSM induces CXCL12 in BM stromal cells.^7^ This is not aligned with results obtained in *Osmr*^−/−^ mice,^9^ which did show a reduction of CXCL12 in the BMEF, possibly because OSM can signal through the LIF receptor to activate STAT3 and potentially sustain the expression of CXCL12.^16^

Aged neutrophils (CD11b^brigHT^Gr-1^bright^CXCR4^high^CD62L^low^, Figure S3), which are supposed to regulate HSPC traffic,^11^ displayed the same circadian levels in PB (Figure 2F) and BM (Figure 2G), which was unaffected by the loss of Osm. In the lung, an important neutrophil margination compartment, *Osm*^−/−^ mice showed an expanded pool of neutrophils at Zt5 (Figure S4), confirming that neutrophils can follows tissue-specific dynamics.^17^

The expression of key clock genes (*Per1* and *Rev-ERBα*) in the BM followed the same trend in Wt and *Osm*^−/−^ mice (Figure 2H). As the homing of aged neutrophils back to the BM was previously found to elicit macrophage phagocytosis,^11^ we also examined phagocytosis genes. The circadian trend of *Mertk* and *Abca1* gene expression in the BM was similar between Wt and *Osm*^−/−^ mice (Figure 2H). In summary, loss of *Osm* resulted in persistently elevated PB-HSPCs with parallel reduction of BM-HSPCs, without affecting the circadian regulation of neutrophils, clock gene expression, and phagocytosis gene expression.

### Neutrophils regulate HSPC kinetics

**Figure 3:**
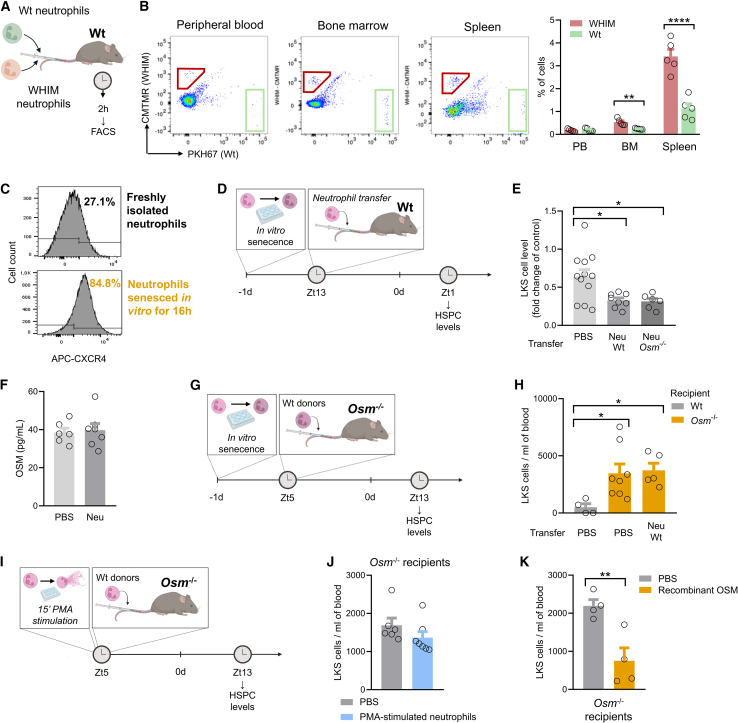
Transferred neutrophils migrate to the BM and regulate HSPC kinetics (A) Competitive transfer of green (PHK67)-labeled wild-type (Wt) neutrophils and red (CMTMR)-labeled WHIM neutrophils into Wt recipients. (B) Wt (green) or WHIM (red)-labeled cells in the blood, bone marrow and spleen of recipient mice, examined by flow cytometry 2 h after injection. Left part, representative FACS analysis, right part, cell levels of the t wo-donor type in the various recipient compartments. (C) Representative flow cytometry histograms showing that *in vitro* aging of bone marrow (BM)-derived neutrophils increases CXCR4 expression. (D) Experimental plan: bone marrow-derived neutrophils from wild-type mice were aged *in vitro* and injected into wild-type mice according to the displayed time schedule. (E) HSPC (Lin^-^cKit^+^Sca1^+^ cells) levels at zenith time (Zt1) after injection of aged Wt or *Osm*^−/−^ neutrophils into Wt recipients at neutrophil nadir (Zt13). Data are presented as fold-change versus baseline to account for the dilution effect that can occur after injections (as a result the control PBS-injected mice have a value below 1.0). (F) OSM concentrations in the bone marrow extracellular fluid (BMEF) of Wt mice injected with vehicle or Wt aged neutrophils. (G) Experimental plan: bone marrow-derived neutrophils from wild-type mice were aged *in vitro* and injected into *Osm*^−/−^ mice according to the displayed time schedule. (H) HSPCs levels after injection of OSM-secreting Wt neutrophils or vehicle (PBS) into in *Osm*^−/−^ recipient mice. The control level of HSPCs in Wt mice injected with PBS is also shown. (I) Experimental plan: bone marrow-derived neutrophils from wild-type mice were stimulated with PMA for 15 min *ex vivo* and then injected into *Osm*^−/−^ mice according to the displayed time schedule. (J) HSPCs levels after injection of PMA-stimulated neutrophils or vehicle (PBS) into in *Osm*^−/−^ recipient mice. (K) HSPC levels in wild-type *Osm*^−/−^ mice injected with PBS or recombinant OSM. Mean ± standard error is displayed in the plots. ∗*p* < 0.05; ∗∗*p* < 0.01; ∗∗∗∗*p* < 0.0001 by two-way ANOVA (B), by one-way ANOVA (E, H) or by Student’s *t* test (F, J, K). n = 4–12 biological replicates for each strain.

It has been shown that interfering with neutrophil clearance by injecting neutrophils can perturb the kinetic of HSPCs and their levels in PB.^11^ We hypothesized that neutrophils returning to the BM can regulate HSPC traffic by secreting OSM. To validate the neutrophil transfer model, we first verified that at least part of injected neutrophils homed to the BM. To this end, we injected the same amount (2×10^6^ cells) of green (PKH67)-labeled Wt neutrophils and red (CMTMR)-labeled WHIM neutrophils simultaneously into Wt recipients at Zt5 (Figure 3A). Neutrophils from WHIM mice harbor a dominant mutation in the *Cxcr4* gene, resulting in a defective recycling upon binding of CXCL12, and thus increased responsiveness to CXCL12 stimulation and propensity to homing. As early as 2 h after injection, labeled neutrophils were found in the BM more than in the PB, and even more so in the spleen. Notably, this was due to active CXCR4/CXCL12-mediated recruitment from the blood to the BM because the percentages of WHIM neutrophils in the BM and spleen were significantly higher than the percentages of Wt neutrophils (Figure 3B).

We then used in vitro-senesced neutrophils, which have an increased expression of CXCR4 (Figure 3C) making them more prone to migrate back to the BM. The adoptive transfer of 2 × 10^6^ Wt neutrophils senesced for >8 h into Wt mice at ZT13 (i.e., at neutrophil nadir) significantly reduced HSPC levels at ZT1 (i.e., HSPCs zenith), thereby confirming that aged neutrophils regulate the HSPC rhythm (Figures 3D, 3E, and S5). Such effect of neutrophils does not appear to be mediated by OSM, as senesced *Osm*^−/−^ neutrophils (Figure S1) transferred into Wt mice were still able to reduce HSPC levels (Figure 3E) and the concentration of OSM in the BMEF was unaffected by injection of Wt senesced neutrophils (Figure 3F).

### Neutrophil-derived OSM does not retain HSPCs in the niche

We thus moved to *Osm*^−/−^ mice, which display persistently elevated HSPCs, and we tested whether HSPC levels can be reduced by the adoptive transfer of Wt senescent (OSM-secreting) neutrophils at ZT5, when neutrophils physiologically start to home back to the BM (Figure 3G). *Osm*^−/−^ mice injected with senescent Wt neutrophils had the same level of HSPCs as did control *Osm*^−/−^ mice injected with vehicle, and both groups of mice still had markedly higher HSPC levels than PBS-injected Wt mice (Figure 3H). The same experiment was repeated with fresh Wt neutrophils stimulated *ex vivo* with PMA for 15 min (Figure 3I), which preserved cell viability (Figure S4), while robustly inducing *Osm* gene expression and OSM release (Figures 1F and S6). When injected into *Osm*^−/−^ recipients, again they failed to reduce HSPCs toward normal levels (Figure 3J). Therefore, OSM-secreting neutrophils failed to provide OSM to the BM and retain HSPCs in the niche. On the other side, injection of recombinant OSM into *Osm*^−/−^ mice significantly reduced HSPC levels (Figure 3K).

**Figure 4:**
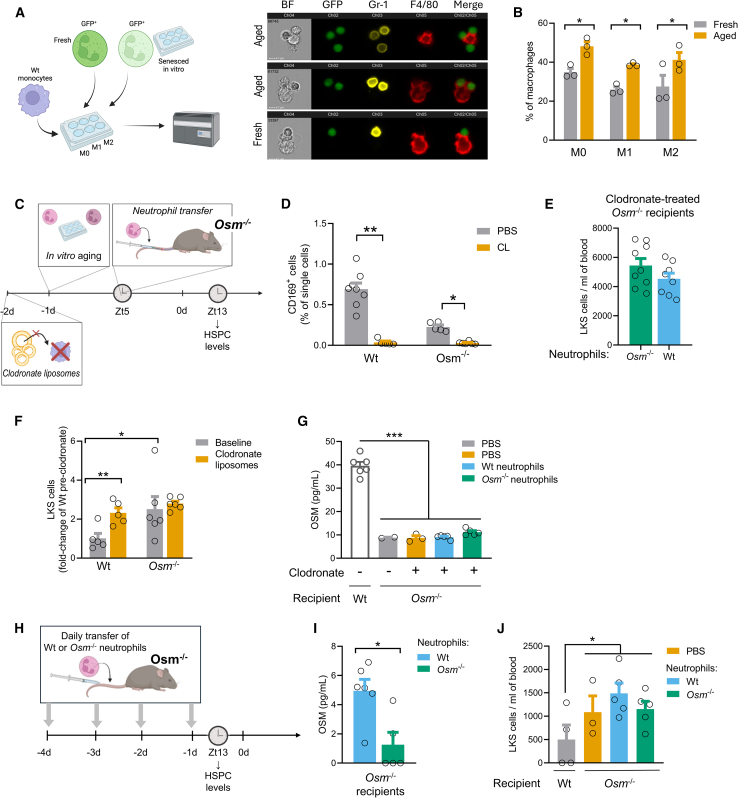
Neutrophil-derived OSM fails to regulate HSPC traffic (A) Representative imaging flow cytometry of GFP^+^ neutrophils (either fresh or aged *in vitro* for 16h) incubated with F4/80 APC-Cy7-labeled BM-derived macrophages. The overlap of green (Ch2, GFP) and red (Ch4, CMTRM) shows neutrophils engulfed in macrophages (far right column). BF, bright field. (B) Percentage of M0, M1, or M2 macrophages with engulfed fresh or aged neutrophils, as determined by conventional flow cytometry. (C) Experimental plan: treatment with clodronate liposomes was used to deplete macrophage at day 2. At day 1 neutrophils were collected for *in vitro* aging. Neutrophils from wild-type or *Osm*^−/−^ donors were injected into *Osm*^−/−^ recipients at Zt5 and HSPC levels were examined at Zt13. (D) Bone marrow CD169^+^ macrophage content in mice treated with clodronate liposomes. (E) HSPC levels in *Osm*^−/−^ mice injected with Wt or *Osm*^−/−^ neutrophils after macrophage depletion with clodronate liposomes. (F) HSPCs levels in Wt and *Osm*^−/−^ mice after macrophage depletion with clodronate liposomes. (G) OSM concentrations in the bone marrow extracellular fluid (BMEF) of wild-type (Wt) or *Osm*^−/−^ mice with or without pre-treatment with clodronate liposomes and injected with PBS, or neutrophils from Wt or *Osm*^−/−^ donors. (H) Experimental plan: neutrophils from Wt or *Osm*^−/−^ mice were injected daily for 4 consecutive days into *Osm*^−/−^ recipients and HSPC levels were examined at Zt13. (I) OSM concentrations in the bone marrow extracellular fluid (BMEF) of *Osm*^−/−^ mice after receiving multiple injections of neutrophils from Wt or *Osm*^−/−^ donors. (J) LKS cell levels in Wt mice and in *Osm*^−/−^ mice after PBS or multiple injection of neutrophils from Wt or *Osm*^−/−^ donors. Mean ± standard error is displayed in the plots. ∗*p* < 0.05; ∗∗*p* < 0.01; ∗∗∗*p* < 0.001 by two-way ANOVA (B-D-F), by one-way ANOVA (G-J) or by Student’s *t* test (E-I). n = 4–9 biological replicates for each strain.

To explain this paradox, we hypothesized that neutrophils homed to the BM failed to secrete OSM because they are rapidly phagocytosed by macrophages. Using imaging flow cytometry, we confirmed that GFP^+^ neutrophils, either fresh or senesced *in vitro* for 16h, can be engulfed by macrophages (Figure 4A). Using conventional flow cytometry, we found that senesced neutrophils were significantly more prone to be phagocytosed than fresh neutrophils, irrespectively of the M0, M1 or M2 polarization status of macrophages (Figure 4B). This phenomenon was also evident *in vivo*, as in Wt mice a small proportion of BM macrophages engulfed injected Wt neutrophils labeled with PKH67 (Figure S7). Therefore, we depleted BM-macrophages using clodronate liposomes before transferring neutrophils (Figure 4C). Even if macrophage fragmentation during preparation can complicate interpretation,^15^ after an apparent effective depletion of CD115^−^Gr-1^-^F4/80^+^SSC^low^CD169^+^ BM macrophages (Figure 4D), *Osm*^−/−^ mice injected with Wt neutrophils at Zt5 had the same HSPC level at Zt13 as did control *Osm*^−/−^ mice injected with *Osm*^−/−^ neutrophils (Figure 4E) and both groups still had very high blood HSPCs. Of note, BM macrophages are a known source of OSM,^7^ and their depletion with clodronate liposomes significantly elevated PB-HSPC levels in Wt but not in *Osm*^−/−^ mice (Figures 4F and S8). This confirms that this raise in HSPCs relies on the reduction of OSM caused by macrophage depletion because such effect was not observed in mice genetically lacking *Osm*.

Second, we hypothesized that a single injection of neutrophils is unable to recapitulate the physiologic recirculation of these cells in the BM. Indeed, OSM concentrations in the BMEF of *Osm*^−/−^ mice could not be raised after a single injection of Wt neutrophils, even after depletion of macrophages (Figure 4G). Therefore, we performed a set of experiments wherein neutrophils from Wt or *Osm*^−/−^ mice were injected daily for 4 consecutive days into *Osm*^−/−^ recipients (Figure 4H). Multiple transfers of Wt neutrophils approach succeeded in raising significantly OSM concentrations in the BMEF compared to the transfer of *Osm*^−/−^ neutrophils (Figure 4I). Probably because of dilution due to multiple injections, HSPC levels tended to be lower than expected in *Osm−/−* mice than in previous experiments, but still they were significantly (*p* = 0.018) higher than in Wt mice. Despite this, the multiple injection of OSM-producing neutrophils in *Osm*^−/−^ mice failed to lower HSPC levels compared to the injection of PBS or *Osm*^−/−^ neutrophils (Figure 4J).

### Neutrophil depletion mobilizes HSPCs

**Figure 5:**
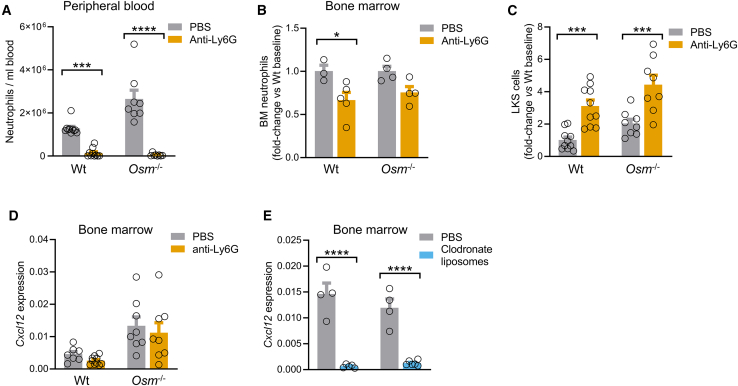
Neutrophil depletion mobilizes HSPCs (A) Depletion of peripheral blood neutrophils with anti-Ly6G antibodies in wild-type (Wt) and *Osm*^−/−^ mice. (B) Depletion of bone marrow neutrophils with anti-Ly6G antibodies in Wt and *Osm*^−/−^ mice. (C) HSPC (Lin^-^cKit^+^Sca1^+^ cells) levels in Wt and *Osm*^−/−^ mice treated with anti-Ly6G antibodies or PBS control. (D) Gene expression of *Cxcl12* in the bone marrow of Wt and *Osm*^−/−^ mice treated with anti-Ly6G antibodies or PBS. (E) Gene expression of *Cxcl12* in the bone marrow of Wt and *Osm*^−/−^ mice treated with clodronate liposomes or PBS. Mean ± standard error is displayed in the plots. ∗*p* < 0.05; ∗∗∗*p* < 0.001; ∗∗∗∗*p* < 0.0001 by two-way ANOVA (A-B-C-D-E).

Finally, we tested a strategy of neutrophil depletion by treating mice with anti-Ly6G antibodies. This was expected to yield the opposite effect of neutrophil transfer, i.e., an increase in PB-HSPC levels. The approach was effective in depleting neutrophils in peripheral blood (Figure 5A) and, to a lesser extent, in the BM (Figure 5B). As expected, neutrophil depletion resulted in a significant 3-fold increase in PB-HSPCs (Figure 5C). Notably, neutrophil depletion raised HSPCs also in *Osm*^−/−^ mice (Figures 5A–5C and S8), suggesting that the mechanisms for this effect does not rely on the suppression of OSM levels. Consistently, there was no modulation of *Cxcl12* expression in the BM (Figure 5D), contrary to what observed for macrophage depletion with clodronate liposomes (Figure 5E) suggesting that the mechanism whereby neutrophils regulate HSPC egress does not converge to the CXCL12/CXCR4 axis downstream of OSM.

## Discussion

OSM is a major player in the regulation of HSPC traffic in/out the BM. Herein, we show that despite the fact that neutrophils exhibit high levels of *Osm* expression and can release the protein, OSM is dispensable for their ability to modulate the egress of HSPCs in unstimulated conditions.

We found that neutrophils that have aged in the bloodstream return to the BM and retain HSPCs in the niche, thereby reducing their levels in PB. This confirms prior observations that the kinetics of neutrophil release and recycle is linked to the rhythmic traffic of HSPC into the circulation.^11^ Such mutual coordination between immune function and hematopoietic activity is instrumental to maintaining ready immune cell pools and mount effective responses to diverse pathologic conditions. COVID-19 is a striking example characterized by severe neutrophilia and profound pauperization of circulating HSPCs. Notably, both a high neutrophil-to-lymphocyte (N/L) ratio,^18^ which is a sign of excess myelopoiesis, and a reduced PB-HSPC count were strong predictors of adverse outcomes in COVID-19.^19^^,^^20^ Of interest, OSM has been identified as part of the hyperinflammatory signature of COVID-19 correlated with disease severity.^21^^,^^22^ OSM is at the same time a driver of myelopoiesis and a retaining factor for HSPCs in the BM,^23^ thereby sustaining a vicious cycle of inflammation. On these bases, OSM was considered an ideal candidate neutrophil-derived factor regulating HSPC traffic.

Despite such role has been initially identified for macrophage-derived OSM,^7^ our new data indicate that neutrophils are the BM and PB cell type expressing the highest level of *Osm* gene. We also provide evidence that neutrophils release OSM, especially when senesced *in vitro* or primed with PMA. While OSM release by neutrophils appeared not to require NETosis, whether it is an active process or occurs upon cell death remains unclear.

This provided a rationale for examining whether aged neutrophils release OSM once they home back to the BM and are removed, thereby regulating the HSPC niche. To verify such hypothesis, we relied on adoptive neutrophil transfer, which was preliminarily validated using the WHIM model. WHIM mice (a model for the human disease featuring *W*arts, *H*ypogammaglobulinemia, *I*nfections, and *M*yelokathexis) harbor a gain-of-function mutation making CXCR4 hyperactive and are characterized by blood neutropenia due to defective neutrophil trafficking.^24^ This model enabled us to demonstrate that injected neutrophils actively home back to the BM via CXCR4 signaling, as WHIM neutrophils reached extravascular sites at significantly higher rates than Wt neutrophils. In fact, homing to the BM is believed to occur when neutrophils become senescent, a condition that increases both surface CXCR4 and *Osm* gene expression.

However, the first evidence that OSM may not be mediating the effects of neutrophils on HSPC levels was the observation that the transfer of *Osm*^−/−^ neutrophils into Wt mice was still able to reduce PB-HSPC levels at their circadian zenith. Nonetheless, this proof was not considered conclusive because transferred neutrophils contributed marginally to the neutrophil pool of Wt recipients, which expressed OSM. In fact, considering that an adult mouse femur in steady state conditions contains approximately 8 × 10^6^ Gr1^+^ neutrophils^25^ and that one femur represents about 5.6% of the total BM of an adult mouse,^26^ there can be up to 140 × 10^6^ BM neutrophils in an adult mouse. Therefore, the number of transferred neutrophils may be too small (1/70 of the body’s neutrophil pool) to exert a sizable biological effect unless they are provided with a key function not shared by host neutrophils. We therefore moved to evaluate whether the transfer of Wt neutrophils, thanks to their expression and release of OSM, could reduce (and so “rescue”) the chronically elevated PB-HSPC levels of *Osm*^−/−^ recipients. This would therefore mimic the effect of injecting recombinant OSM directly into *Osm*^−/−^ mice, which was effective in lowering PB-HSPCs. However, the transfer of aged or PMA-activated Wt neutrophils failed to reduce PB-HSPCs in *Osm*^−/−^ mice. As we found that a single neutrophil transfer did not raise OSM concentrations in the BMEF to a detectable level, we hypothesized that homed neutrophils could be removed by BM macrophages. Yet, even after pre-emptive depletion of BM macrophages, the transfer or Wt neutrophils failed to reduce PB-HSPCs in *Osm*^−/−^ mice. As a limitation of this approach, we wish to acknowledge that the use of clodronate liposomes to deplete macrophages may result in stunning of neutrophils,^27^ thus confounding the interpretation. We thus pursued an approach of multiple transfer of neutrophils into *Osm*^−/−^ mice. Despite a significant increase in OSM concentrations in the BMEF, this strategy also failed to lower HSPC levels, which remained elevated. Therefore, neutrophil-derived OSM seems unable to regulate the HSPC niche, their traffic and PB levels.

We can speculate on the reasons why injecting recombinant OSM was effective in reducing HSPCs in *Osm*^−/−^ mice while injecting Wt neutrophils was not. First, the achieved concentrations of OSM in the BM might be insufficient to reverse the effect of a chronic absence of OSM, Second, neutrophils may release OSM only under some circumstances, such as aging or cell death. Third, in a time- and space-compartmentalized niche,^28^ neutrophil-derived OSM may not effectively come into contact with HSPCs.

On the other side, we have confirmed that depletion of BM macrophages in Wt mice induced the release of HSPCs into the PB,^29^ which is believed to rely on the suppression of OSM production within the BM niche.^7^ Indeed, the effect was not observed in *Osm*^−/−^ mice, that already display the maximum elevation in PB-HSPC levels that can be attributable to the absence of OSM. We now show that neutrophil depletion exerted an effect on PB-HSPC that was opposite to that obtained with neutrophil transfer, i.e., an elevation in PB-HSPCs. Notably, this effect does not appear to rely on the suppression of OSM in the BM niche, because the same effect was present in *Osm*^−/−^ mice on top of their chronic elevation in PB-HSPCs. In summary, our complementary set of experiments suggest that neutrophils regulate the traffic of HSPCs in an OSM-independent pathway. Differently from macrophages, that are constitutive part of the BM niche, waves of neutrophils in the BM due to circadian rhythms or pathologic conditions may regulate HSPC migration with mechanisms not converging on the CXCL12/CXCR4 axis.

Our conclusion contrasts with prior evidence suggesting that aged neutrophils are cleared by BM macrophage efferocytosis, generating LXR-dependent, but otherwise undefined, signals that downregulate CXCL12 in the BM, thereby promoting HSPC egress.^30^ In fact, several HSPC-mobilizing actions of neutrophils are known to be independent from the regulation of CXCL12, such as the loosening of cell-matrix interactions by metalloproteinases,^31^^,^^32^ the generation of reactive oxygen species,^33^ and the production of PGE_2_.^34^ These events can be involved in the mobilization response to G-CSF. As neutrophil depletion with anti-Gr1 antibodies reduced both HSPC mobilization and BM OSM concentrations,^9^ it is still possible that neutrophil-derived OSM counters HSPC release after stimulation with G-CSF.

Our study helps further understand the complexity of the BM niche, illustrating how the cell-specific origin of OSM affects its ability to modulate HSPC traffic. Indeed, niche-modulating factors are typically compartmentalized and constrained in time and space to regulate HSPC quiescence versus activity, cell fate decisions, and migration versus homing. Strategies aimed at targeting the hematopoietic effects of OSM should take this newly discovered context-dependent signaling into consideration.

### Limitations of the study

This study has some limitations. First, we used adoptive transfer of neutrophils to explore their regulatory role, but this approach may not fully mimic the physiological recirculation of neutrophils that occurs *in vivo*. Second, *Osm*^−/−^ mice may activate compensatory pathways that partially mask the effects of OSM on HSPC trafficking. To address this, targeted and conditional knock-out models that specifically delete Osm in neutrophils and macrophages/monocytes could offer deeper insight.^35^ Although the BM is a key site for neutrophil clearance, the liver and spleen also contribute to their removal from circulation but were not addressed in this study. Finally, clodronate liposomes deplete macrophages systemically, potentially causing off-target effects in other tissues.

## Resource availability

### Lead contact

Further information and requests for resources and reagents should be directed to and will be fulfilled by the lead contact, Gian Paolo Fadini (gianpaolofadini@unipd.it).

### Materials availability

This study did not generate new unique reagents.

### Data and code availability

• Data reported in this paper will be shared by the lead contact upon request.
• This paper does not report original code.
• Any additional information required to reanalyze the data reported in this paper is available from the lead contact upon request.

## Acknowledgments

This study was supported by grants from the 10.13039/501100001648European Foundation for the Study of Diabetes (2016 Lilly Research Fellowship Application), the Italian Ministry of University (PRIN projects 201793XZ5A and 2022MZTHWJ and PRIN project 202077EYN7 Finanziato dall’Unione europea- Next Generation EU, Missione 4 Componente 1, CUP 202077EYN7_001 to G.P.F.), the 10.13039/501100003500University of Padova (DOR; STARS@UNIPD DiaNETes to G.P.F.; 2021 STARS@UNIPD Finanziato dall’Unione Europea NextGenerationEU “MalTraDiates” to M.A.) and as part of the activities of the National Center for Gene Therapy and Drugs Based on RNA Technology, funded in the framework of the National Recovery and Resilience Plan (NRRP), Mission 4, Component 2, Investment 1.4, funded by the European Union - Next Generation EU, Project CN00000041, CUP C93C22002780006, Spoke n. 4 “Metabolic and Cardiovascular diseases”. Images were generated using Biorender software (Biorender.com).

## Author contributions

Conceptualization, M.A. and G.P.F.; methodology A.R., C.B., F.I.A., L.M., and M.A; investigation, A.R., C.B., F.I.A., L.M., and M.A.; writing – original draft, M.A. and G.P.F.; resources A.V. and B.M.; writing – review and editing, M.A., C.B., and G.P.F; visualization, A.R., C.B., M.A., and G.P.F.; funding acquisition, M.A. and G.P.F.; supervision M.A. and G.P.F.

## Declaration of interests

M.A. and G.P.F. are the inventors of a patent to use OSM inhibition for stem cell mobilization (WO2016046738A1). The authors have nothing else to declare.

## STAR★Methods

### Key resources table

**Table:** 

REAGENT or RESOURCE	SOURCE	IDENTIFIER
**Antibodies**		

Lineage Cocktail(CD3, Ly-6G/C, CD11b, CD45R, TER-119) Pacific Blue	BioLegend	Cat# 133306, RRID: AB_11126978
Isotype control	BioLegend	Cat# 133306, RRID: AB_11126978
Rat Anti-Mouse CD117 (c-kit) FITC	eBioscience	Cat# 11-1171-85, RRID: AB_465187
Rat Anti-Mouse Ly-6A/E (Sca-1) PE	eBioscience	Cat# 12-5981-83, RRID: AB_466087
Rat Anti-Mouse F40-80 APC/Cy7	BioLegend	Cat# 123118, RRID: AB_893477
Rat Anti-Mouse Ly-6G/C (Gr-1) PE	eBioscience	Cat# 108408, RRID: AB_313373
Rat anti Anti-Mouse Ly6G FITC	eBioscience	Cat# 551460, RRID: AB_394207
Rat Anti-Mouse CD11b PE-Cy7	eBioscience	Cat# 101216, RRID: AB_312799
Rat Anti-Mouse Ly6C eFluor™450	eBioscience	Cat# 48-5932-82, RRID: AB_10805519
Rat Anti-Mouse Siglec-F Brilliant Violet 421™	BioLegend	Cat# 155509, RRID: AB_2810421
Rat Anti-Mouse CD16/32 (Fc block)	BD Biosciences	Cat# 553142, RRID: AB_394657
Anti-mouse CD115 (CSF-1R) AlexaFluor™ 488	Thermo Fisher Scientific	Cat# 135512, RRID: AB_11218983
Anti-mouse CD62L PerCP/Cy5.5	Thermo Fisher Scientific	Cat# 104432, RRID: AB_2285839
Anti-mouse CD184 (CXCR4) APC	Thermo Fisher Scientific	Cat# 17-9991-82, RRID: AB_10670878
Recombinant mouse IgG2a anti Ly-6G antibody	absolute antibody	Cat# Ab00295–2.0
PerCP/Cyanine5.5 anti-mouse CD45 Antibody	Biolegend	Cat# 103132, RRID: AB_893340

**Chemicals, peptides, and recombinant proteins**		

phorbol 12-myristate 13-acetate (PMA)	Merck	Cat#P1585
Calcium Ionophore A23187	Merck	Cat# C7522
LPS from Escherichia coli (O55:B5)	Merck	Cat#L2880
Doxorubicin	Selleckchem	Cat# E2516
PKH26 membrane labeling	Merck	Cat# PKH26GL-1KT
CellTracker™ Orange CMTMR	Thermo Fisher	Cat# C2927
Recombinant Mouse Oncostatin M (OSM) Protein	Bio Techne	Cat# 495-MO-025/CF
Clodronate Liposome	Liposoma BV	Cat# CP-005-005
Recombinant Mouse Interleukin-4 (rm IL-4)	ImmunoTools	Cat# 12340043
Recombinant Mouse Interleukin-13 (rm IL-13)	ImmunoTools	Cat# 12340133
Recombinant Mouse Interferon/γ (rm IFN-γ)	ImmunoTools	Cat# 12343536
Recombinant Mouse Macrophage Colony Stimulating Factor (rm M-CSF)	ImmunoTools	Cat# 12343115
QIAzol Lysis Reagent	Qiagen	Cat# 79306

**Critical commercial assays**		

Mouse Neutrophil Isolation Kit	Miltenyi Biotec	Cat# 130-097-658
MACSxpress® Whole Blood Neutrophil Isolation Kit, human	Miltenyi Biotec	Cat# 130-104-434
FITC Annexin V Apoptosis Detection Kit I	BD Pharmingen™	Cat# 556547 RRID: AB_2869082
Mouse Oncostatin M (OSM) DuoSet ELISA	Bio-Techne	Cat# DY495-05
Mouse CXCL12/SDF-1α ELISA Kit - Quantikine	Bio-Techne	Cat# MCX120
CellEvent™ Senescence Green Flow Cytometry Assay Kit	Thermo Fischer	Cat# C10840

**Deposited data**		

Tabula Muris	https://tabula-muris.ds.czbiohub.org/	GSE109774
Tabula Sapiens	https://tabula-sapiens.sf.czbiohub.org/	GSE201333

**Experimental models: Organisms/strains**		

C57BL/6J mice	Jackson Laboratory	Cat# 000664
Mouse: B6(Cg)- Padi4^tm1.2Kmow^/J	Jackson Laboratory	Cat# 026708, RRID:IMSR_JAX:026708
Mouse: B6(Cg)-*Commd10*^*Tg(Vav1-icre)A2Kio*^/J	Jackson Laboratory	Cat# 008610, RRID:IMSR_JAX:008610
Mouse: CXCR4^+/1013^ knock-in	Institut Pasteur	N/A
Mouse: Osm^−/−^ mice	GlaxoSmithKline	N/A

**Software and algorithms**		

GraphPad Prism 9.0	Dotmatics	RRID:SCR_002798
FlowJo software v10.10	BD	RRID:SCR_008520

### Experimental model and study participant details

#### Human samples

Human neutrophils were isolated from the peripheral blood of healthy blood donors using the MACSxpress Human Neutrophil Isolation kit (Miltenyi Biotec, Germany) according to the manufacture’s instruction, while PBMCs were collected by density gradient centrifugation of peripheral blood with Histopaque-1077 (Merck, Germany) at 330g for 30 min and collected at the interface with plasma. The protocol for blood collection from anonymous healthy donors was approved by the Ethical Committee of the University Hospital of Padua and the subjects provided informed consent (Prot. 879, 09/01/2015).

#### Animals

For all experiments, we used three to five-month-old male and female animals on a C57BL/6J background randomly assigned to the experimental groups. Mice were housed with a maximum of 5 animals per cage with access to food and water *ad-libitum* and with environmental enrichments. The animals were kept at 23° in constant 12-h dark/light cycle where the lights turn on at 07:00 a.m. (Zt0). All studies were performed at the Veneto Institute of Molecular Medicine (authorization number 175/2002A) and were approved by the Veneto Institute of Molecular Medicine Animal Care and Use Committee and by the Italian Health Ministry (authorizations n° 128_2018-PR - A06E0.12 and 29_2018-PR - A06E0.13). C57BL/6J (Wt) and C57BL/6-Tg(UBC-GFP) mice were purchased from The Jackson Laboratory and established as a colony since 2001 and 2017, respectively. Hemizygous B6(Cg)- Padi4tm1.2Kmow/J (Strain #026708, RRID:IMSR_JAX:026708, Padi4^+/fl^), B6(Cg)-Commd10Tg(Vav^1−icre^)A2Kio/J (Strain #008610, RRID:IMSR_JAX:008610, Vav1^+/Cre^) mice were purchased from The Jackson Laboratory established as a colony 2018. C57BL/6J *Osm*^−/−^ mice were obtained from GlaxoSmithKline (Stevenage, U.K.). WHIM-associated mutant CXCR4^+/1013^ knock-in mice (WHIM mice) were generated at the Institut Pasteur^36^ and kindly provided by Dr. F. Arenzana-Seisdedos. Additional information can be found in the Appendix.

### Method details

#### Peripheral blood and BMEF

Peripheral blood of mice was collected at different Zt (zeitgeber time) points by retro-orbital bleeding in EDTA-coated tubes. WBC count and blood formula was performed using the CELL-DYN Emerald (G4-9513/R06, Abbott Laboratories). Plasma was obtained by centrifugation of peripheral blood for 10 min at 2500 rpm. BMEF was isolated by flushing one femur by centrifugation at 13000 rpm for 10 s. The pellet was gently resuspended in 500 μL of sterile ice-cold PBS and kept on ice for 10 min. Cell suspension was centrifuged at 2000 rpm for 10 min and the supernatant (BMEF) was collected and stored at −80°C.

#### Mouse neutrophils

Bone marrow neutrophils were isolated using the Neutrophils Isolation Kit (Miltenyi Biotec). Senescence was induced *ex vivo* by plating 2 × 10^6^/mL neutrophils in RPMI-1640 10% FBS (Corning) with L-glutamine (2 mM) and penicillin/streptomycin for 8 to 16 h. In separate experiments, purified neutrophils were stimulated with 300 nM of PMA (Sigma-Aldrich, Cat. No. P1585, Merck) for 3 h, with 5 μM of A23187 (Merck) for 1 h, or with 1 μM of LPS (Merck) for 1 h in complete medium.

For adoptive transfer experiments, at specific Zt time points each mouse was injected intravenously with 2 × 10^6^ senescent neutrophils and sacrificed at specific Zt time points. For repetitive transfer experiments, 2 × 10^6^ freshly isolated neutrophils were intravenously injected at Zt5 into *Osm*^−/−^ mice for 4 consecutive days. Mice were sacrificed the 5th day at specific Zt time points.

For experiments with WHIM mice, Wt neutrophils were labeled with PKH67 Green Fluorescent Cell Linker (Merck) while WHIM neutrophils were labeled with CellTracker Orange CMTMR (Invitrogen) according to the provided protocols. 2 × 10^6^ neutrophils for each genotype were mixed in a 1:1 ratio and injected intravenously.

Phagocytosis *in vivo* was assessed by injecting at 2 × 10^6^ PKH67-labelled senescent Wt neutrophils at Zt5 and analysing the BM at Zt13.

#### Bone marrow derived macrophages (BMDMs)

Macrophages were differentiated by plating unfractioned BM cells in RPMI-1640, 10% FBS (Corning) with L-glutamine (2 mM) and penicillin/streptomycin with 50 ng/mL rmM-CSF for 7 days with 1 medium change at day 4. At day 7, BMDMs were polarized toward M1 with 500 ng/mL LPS (Merck) and 25 ng/mL IFN-γ or M2 profile with 20/ng/mL IL-4 and 20 ng/mL IL-13 for 48 h. Unless specified, all cytokines were from Immunotools GmbH (Germany).

For phagocytosis experiments, BMDMs were co-cultured with senescent GFP^+^ neutrophils or with CMTMR-labelled neutrophils at 1:2 ratio and collected after 6 and 24 h.

#### ELISA assays

Commercial ELISA kits assays were used to quantify OSM (Cat. No DY495-05, Bio-Techne, Minneapolis, USA) and CXCL12 (Cat. No. MCX120, R&D Systems, Inc, Bio-Techne, Minneapolis, USA) in plasma, BMEF or culture supernatant.

#### Molecular biology

RNA was isolated using the Total RNA Purification Micro Kit (Norgen Biotek) or with QIAzol Lysis Reagent (QIAGEN) and quantified with a NanoDrop 2000 Spectrophotometer (Thermo Fisher Scientific, MA). cDNA was synthesized using the SensiFAST cDNA Synthesis Kit (Bioline, London, UK). qPCR was performed using the SensiFAST SYBR Lo-ROX Kit (Bioline) via QuantStudio 5 Real-Time PCR System (Thermo Fisher Scientific). The list of primers is provided in the Appendix. Target genes were normalized to Ubiquitin (Ubc) and analyzed using the 2^−ΔCt^ method.

#### Flow cytometry and imaging flow cytometry

Single-cell suspensions of BM was obtained by flushing 2 femurs and 2 tibiae with ice-cold PBS through a 70 μm cell strainer and resuspended with 2 mL of MACS. Single cell suspension of the lungs was obtained by enzymatic digestion of minced tissue as described elsewhere.^37^ 200 μL of BM cells, lungs or 100 μL of EDTA-treated blood were incubated with antibodies for 15 min at room temperature. Red blood cells were lysed with standard ACK buffer. Data were acquired with a FACSCanto II (BD Biosciences) cytometer followed by analysis using FlowJo (BD Biosciences). In separate experiments cells were sorted with a FACSCalibur cytometer BD Biosciences). The list of antibodies is provided in the Appendix. Senescence was assessed using the CellEvent Senenescence Green Flow KIT (C10840, Invitrogen) and apoptosis was quantified using the Annexin V Apotosis Detection Kit (Cat# 556547, BD Pharmingen). For imaging flow cytometry, cells were collected and stained and then fixed with 4% œ-formaldehyde for 15 min at RT. Data acquisition was performed with a 6-channel Amnis Image StreamMkII imaging flow cytometer (Cytek Biosciences, Fremont, CA, USA) equipped with 405 nm, 488 nm, and 642 nm lasers. Data were acquired using the integrated software INSPIRE (Cytek Biosciences). The following acquisition settings were applied: brightfield on, 488 nm laser on at a power of 200 mW, low-speed fluidics, magnification at 33 60×, core size of 7 μm, numerical aperture of 0.9, DOF of 2.5 μm. Images were analyzed with IDEAS 6.3 (Image Data Exploration and Analysis Software). Single-color compensation samples were acquired to calculate the spectral crosstalk matrix for spectral compensations in the detection channels. The resulting compensated data files were analyzed using image-based algorithms. Single cells were separated from debris and doublets using a bivariate plot of aspect ratio vs. area of the BF image. Cells in best focus were identified using Gradient RMS feature of the BF image. BMDMs were counterstained with APC-Cy7 anti-F4/80 antibody, while GFP^+^ neutrophils were counterstained with PE anti-Gr-1 antibody. Phagocytosis events were considered when GFP fluorescence were found inside F4/80^+^ BMDMs circumference.

#### *In vivo* treatments

For OSM treatment *in vivo*, mice were injected intraperitoneally with saline or 0.5 μg of carrier-free mouse recombinant OSM (495-MO/CF; R&D Systems, Minneapolis, MN) every 6 h for 48 h. To deplete bone marrow macrophages, mice were intravenously injected with 250 μL of Clodronate Liposomes (Liposoma BV, Amsterdam, The Netherlands) and sacrificed 48 h later. Neutrophils were depleted through intraperitoneal injections of 50 μg of recombinant mouse IgG2a anti Ly-6G antibody (clone 1A8, Absolute Antibody, Oxford, UK) at day 0, 3 and 5. At day 7 mice were sacrificed. Control animals were injected with PBS liposomes and isotype antibody, respectively.

### Quantification and statistical analysis

Single cell RNA sequencing (scRNAseq) data were obtained by using the analysis and visualization tools featured in Tabula Muris and Tabula Sapiens websites, respectively.^12^^,^^13^ All analyses were performed using GraphPad Prism (GraphPad Software, La Jolla, CA). Data are reported as the mean ± SE for continuous variables. Variables were compared between two or more groups using Student’s t test or one-way ANOVA, respectively. For 2 × 2 factorial experiments (e.g., treatment by genotype), the two-way ANOVA was used. Alpha adjustment for multiple comparisons was performed using Bonferroni correction. Results were considered significant at *p* < 0.05. The statistical details of experiments and the number of biological replicates is reported in figure legends, and they are represented as individual data points in graphs.
