# Supplementary material for: Oncostatin M is dispensable for the regulation of hematopoietic stem/progenitor cell traffic by neutrophils
Source: iScience. 2025 May 12;28(6):112646. doi: 10.1016/j.isci.2025.112646 (PMC12158506; doi:10.1016/j.isci.2025.112646)

iScience, Volume *28*

Supplemental information

Oncostatin M is dispensable for the regulation of hematopoietic stem/progenitor cell traffic by neutrophils

Anna Rodella, Carlotta Boscaro, Francesco Ivan Amendolagine, Ludovica Migliozzi, Barbara Molon, Antonella Viola, Mattia Albiero, and Gian Paolo Fadini

Online Appendix

Table S1. List of primers for gene expression analysis.

Gene	Symbol	Sequence
Ubiquitin (housekeeping)	Ubc	Fw: GCCCAGTGTTACCACCAAGA
		Rv: CCCATCACACCCAAGAACA
Oncostatin-M	Osm	Fw: TCAGGGGTCTGATGACACAA
		Rv: AGGGAAAGGAGGAAGTCTGG
C-X-C motif chemokine 12	Cxcl12	Fw: CGGGTCAATGCACACTTGTC
		Rv: GAGCCAACGTCAAGCATCTG
ATP binding cassette subfamily A member 1	Abca1	Fw: TCTGAGAAACACTGTCCTCCTTT
		Rv: TCCTTGGGGACAGAATTGCC
MER Proto-Oncogene Tyrosine Kinase	Mertk	Fw: GAGGACTGCTTGGATGAACTGTA
		Rv: AGGTGGGTCGATCCAAGG
Calprotectin	S100A8	Fw: TCACCATGCCCTCTACAAGA
		Rv: CCAATTCTCTGAACAAGTTTTCG
Prostaglandin-endoperoxide synthase (PTGS-2)	Cox-2	Fw: CATCCCCTTCCTGCGAAGTT
		Rv: CATGGGAGTTGGGCAGTCAT
Interleukin 1-β	Il-1 β	Fw: AGCTTCCTTGTGCAAGTGTCT
		Rv: GACAGCCCAGGTCAAAGGTT

Figure S1. Detection of cellular senescence in mouse neutrophils. Wild type and $Osm^{-/-}$ BM-derived neutrophils were senesced in vitro for 16 hours and positivity for beta-galactosidase was assessed with flow cytometry. Doxorubicin 1 μM was used as positive control.

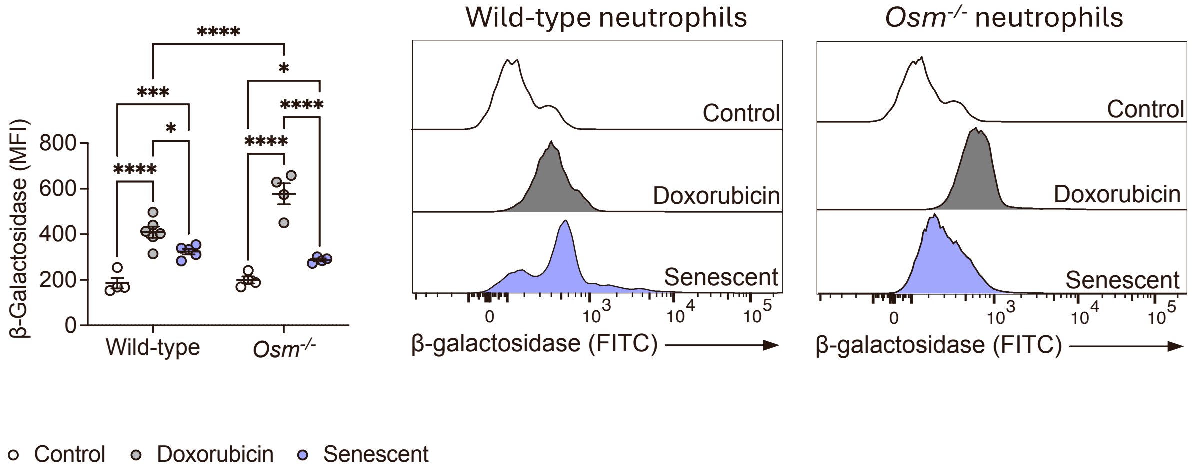

Figure S2. Annexin V/PI staining of freshly isolated BM-derived neutrophils. Wild type or NETosis-deficient Padi4-/- neutrophils were assesed in unstimulated condition, stimulated with PMA, A23158 ionophore and LPS. A). Representative FACS plot of neutrophils among different conditions showing live (white), early apoptotic (light grey), late apoptotic (light blue) and necrotic (light red) cells. B) Histogram showing cells percentages at different stages of apoptosis in Padi4KO and Wt neutrophils among different treatments.

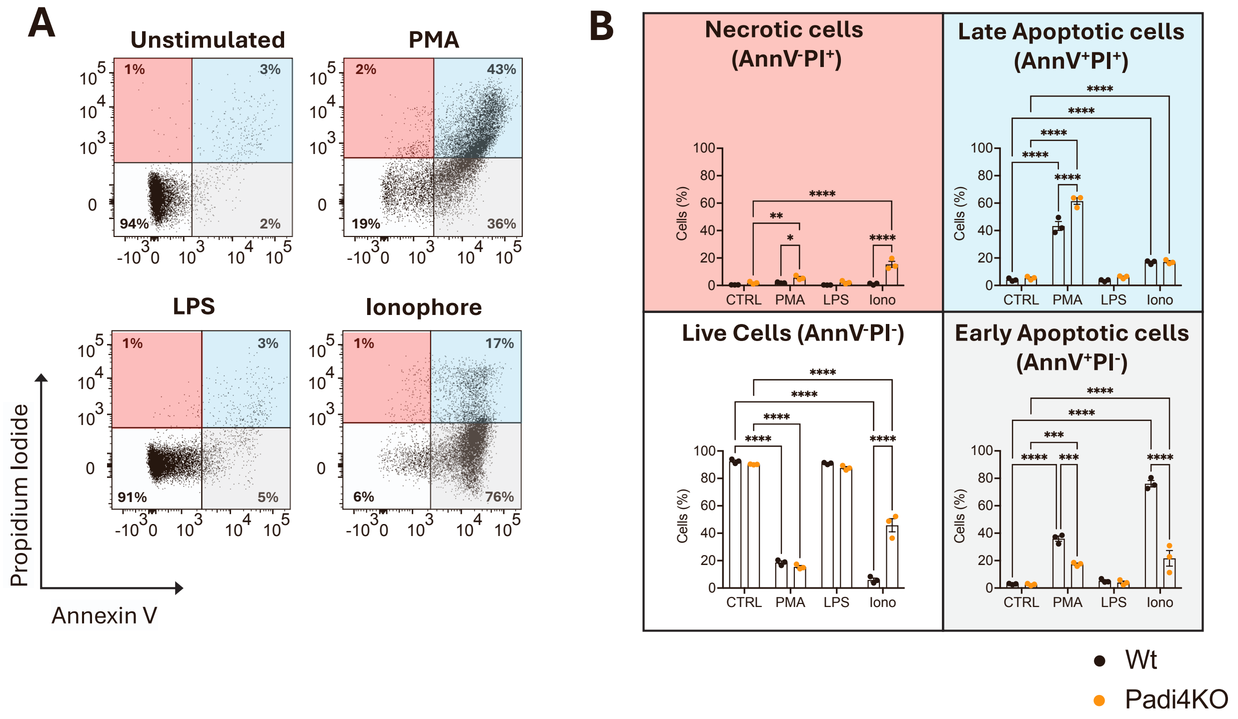

Figure S3. Analysis of aged neutrophils. Representative FACS plot of aged neutrophils at two time points gated according to isotype controls.

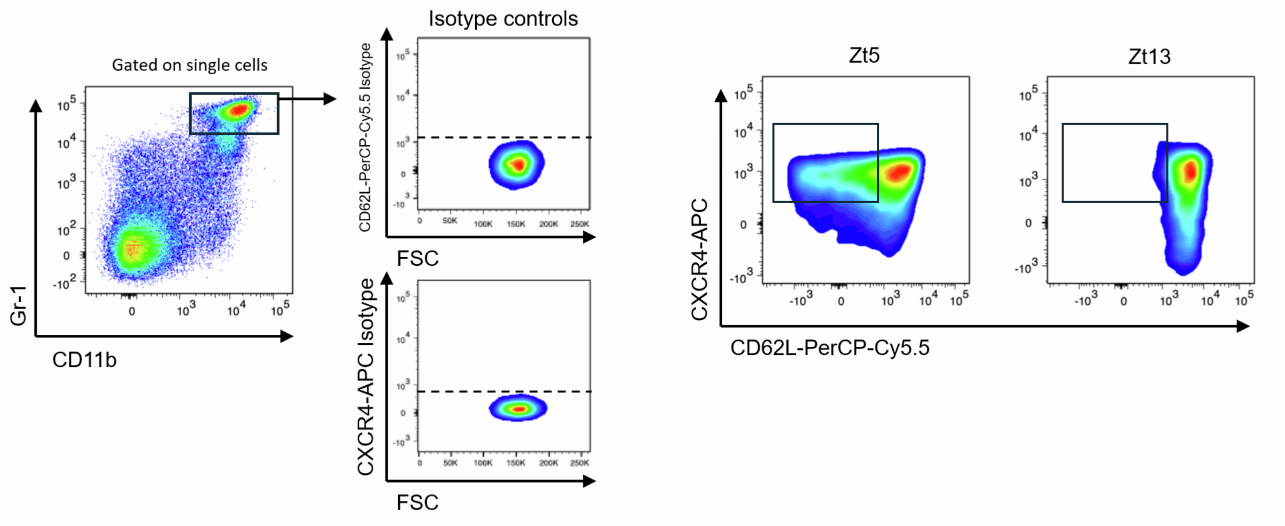

Figure S4. Quantification of myeloid cells subsets in the lung of Wild-type and Osm-/- mice. A) Gating strategy and representative FACS plots of lungs analysed. B) Absolute quantification of different myeloid subsets

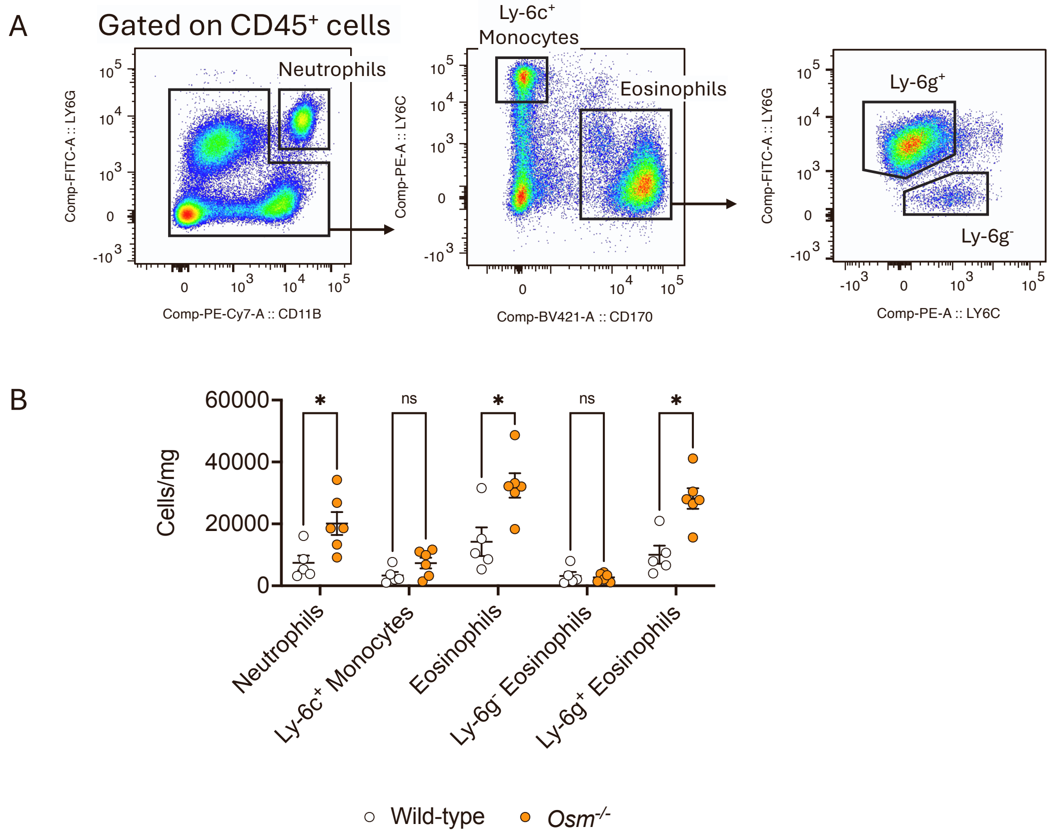

Figure S5. Effect of neutrophils transfer on HSPCs. Representative FACS plot showing the reduction of LKS levels in Wt mice injected with Wt neutrophils versus in control Wt.

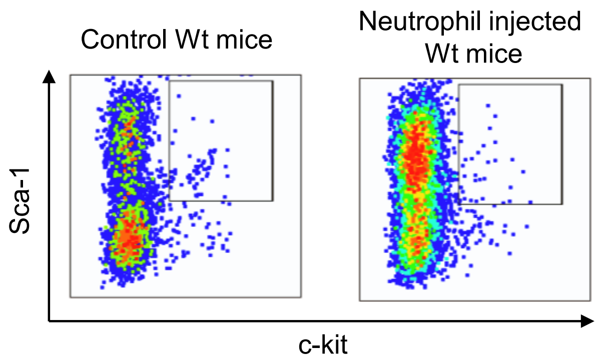

Figure S6. Induction of OSM and viability after PMA stimulation in vitro. Time-dependent increase in Osm gene expression (A) and OSM secretion in the medium (B) when BM-derived Wt neutrophils were stimulated with PMA before adoptive transfer to Osm$^{-/-}$ mice. (C,D) Viability of neutrophils after PMA stimulation assessed with Annexin V/PI staining.

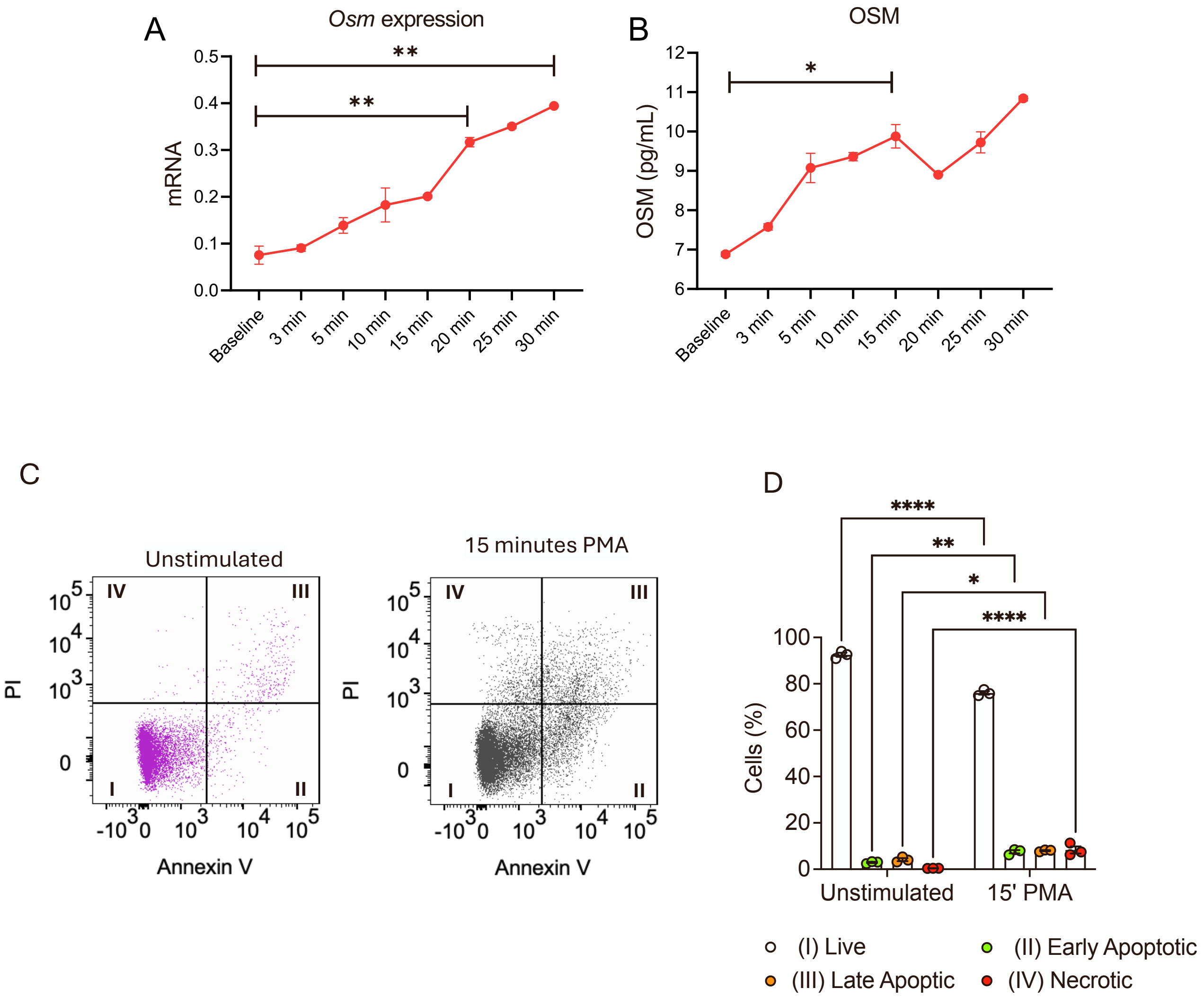

Figure S7. Phagocytosis of injected neutrophils by BM macrophages. A) Identification of PKH67-labelled senescent neutrophils homed to the BM of recipient mice. B) Representative plots of PKH67-labelled senescent neutrophils engulfed by Gr-1^{-}F4/80^{+}CD169^{+} macrophages.

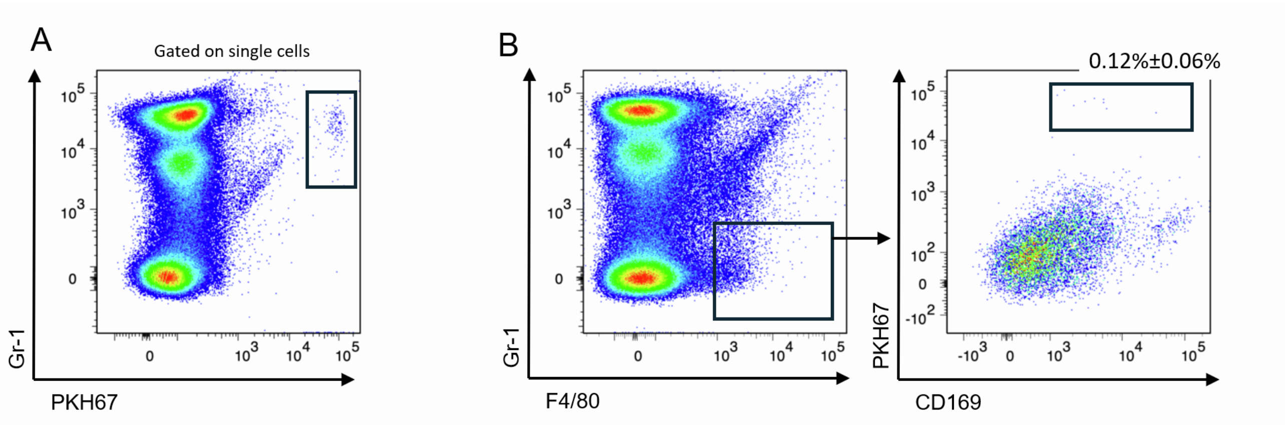

Figure S8. HSPC levels after depletion of macrophages or neutrophils. LKS cell levels in Wt and Osm$^{-/-}$ mice in the control condition and after macrophage (upper part) or neutrophil (lower part) depletion with clodronate liposomes or anti-Ly6G antibodies, respectively.

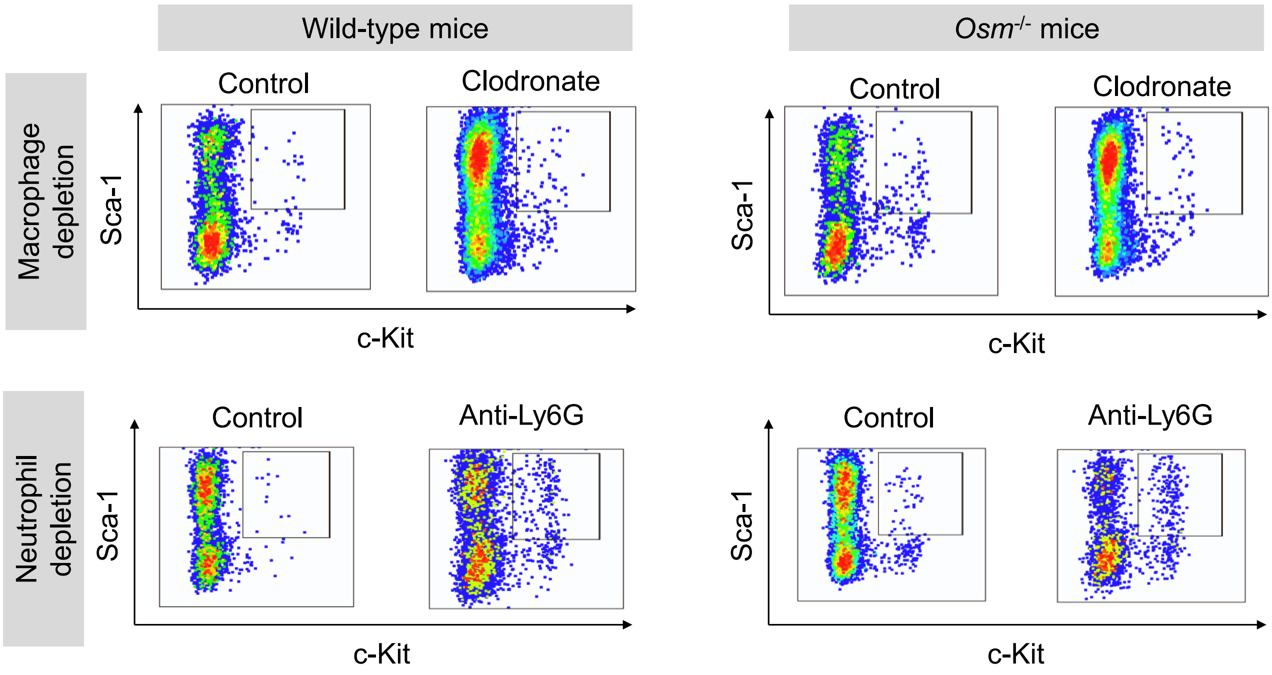

Supplement: Document S1. Figures S1–S8 and Table S1 [file mmc1.pdf]
